# Influence of 12-Week Concurrent Training on Exosome Cargo and Its Relationship with Cardiometabolic Health Parameters in Men with Obesity

**DOI:** 10.3390/nu15133069

**Published:** 2023-07-07

**Authors:** Brisamar Estébanez, Francisco J. Amaro-Gahete, Cristina Gil-González, Javier González-Gallego, María J. Cuevas, David Jiménez-Pavón

**Affiliations:** 1 Institute of Biomedicine (IBIOMED), University of León, 24071 León, Spain; b.estebanez@unileon.es (B.E.); jgonga@unileon.es (J.G.-G.); mj.cuevas@unileon.es (M.J.C.); 2 Department of Physical Education and Sports, Faculty of Sports Science, Sport and Health University Research Institute (iMUDS), 18016 Granada, Spain; amarof@ugr.es; 3 CIBER de Fisiopatología de la Obesidad y Nutrición (CIBEROBN), Instituto de Salud Carlos III, 28029 Madrid, Spain; 4 Instituto de Investigación Biosanitaria, ibs.Granada, 18012 Granada, Spain; 5 MOVE-IT Research Group, Department of Physical Education, Faculty of Education Sciences, University of Cadiz, 11519 Cádiz, Spain; cristina.gil@uca.es; 6 Biomedical Research and Innovation Institute of Cádiz (INiBICA), 11519 Cádiz, Spain; 7 Centro de Investigación Biomédica en Red de Enfermedades Hepáticas y Digestivas (CIBERehd), 28029 Madrid, Spain; 8 CIBER of Frailty and Healthy Aging (CIBERFES), 28029 Madrid, Spain

**Keywords:** concurrent training, cardiometabolic health parameters, exosomes, males, obesity

## Abstract

Exosome release varies depending on the physiological state of the cell, so they could play a fundamental role in obesity, the biggest pandemic in today’s societies. The beneficial effects that physical activity has both on weight and cardiovascular parameters may be mediated by exosomes released in response to exercise. Thus, we aimed (I) to study the influence of a 12-week CT intervention on exosome cargo modifications in men with obesity and (II) to determine whether changes in exosomes after the intervention were related to changes in cardiometabolic health parameters in our cohorts. An experimental, controlled design was performed in twelve (nine with valid data) adult male obese patients (mean values: 41.6 years old, 97.6 kg and 32.4 kg/m^2^) who were randomly divided into a control group (*n* = 4) and a training group (*n* = 5), which completed 36 sessions of CT (concurrent training) for 12 weeks. Before and after the training period, cardiometabolic health parameters were evaluated and blood samples to measure exosomes and proteins were drawn. No changes were observed in the levels of any exosomal markers and proteins; however, associations of changes between CD81 and both fat mass and weight, Flot-1 and VO_2max_, HSP70 and both CRP and left ventricle diastolic diameter or CD14 and leptin were found (all *p* ≤ 0.05). Although the current CT was not able to clearly modify the exosome cargo, a certain medium to large clinical effect was manifested considering the nature of this study. Moreover, the associations found between the promoted changes in cardiometabolic parameters and exosome-carried proteins could indicate a relationship to be considered for future treatments in patients with obesity.

## 1. Introduction

The worldwide prevalence of obesity has almost tripled in the last 40 years, with 39% and 13% of adults now considered to be overweight and have obesity, respectively [1]. Obesity is known to be strongly related with all-cause mortality and is associated with a higher risk of premature mortality [2,3,4]. In addition, obesity is considered a direct risk factor for many diseases including cardiovascular disease (hypertension, coronary heart disease or sudden cardiac death) [5,6] and age-related diseases (dementia, Alzheimer or frailty) [7,8,9,10]. 

Adipose tissue releases a wide range of adipokines as indicators to the organs of the functional status [11]. Thus, the dysregulation of adipose secretome is related to systemic metabolic dysfunction and cardiovascular disease [11]. Recent studies have demonstrated that adipose tissue secretes exosomes, which could influence whole-body metabolism [12]. Exosomes are small extracellular vesicles (30–150 nm) of endocytic origin, formed by endosomal membranes and grouped in multivesicular bodies, that are released from larger microvesicles or apoptotic bodies into circulation so that they reach the target cells with whom they will fuse, establishing cell-to-cell communication [13]. In addition to the paracrine effects, adipose exosomes can act as endocrine factors for the metabolic profiles of different organs, contributing to the organ crosstalk between adipose tissue, liver, skeletal muscle, pancreas and cardiovascular system, among others [14]. In fact, it has been shown that exosomes play a major role in the development of insulin resistance in obesity [15,16]. However, exosome biogenesis has been found to be regulated by adipokines such as adiponectin [17] or glucagon [18].

The benefits of exercise on health and obesity are well-recognized and identified across many organ systems [19,20]. It has been suggested that these effects are partially driven by exerkines, which are defined as signposts released in response to acute/chronic exercise [13,19,20]. Exerkines can be released by numerous cells, tissues and organs (skeletal muscle, heart, liver, white and brown adipose tissues or neurons…); these factors are not only relevant for the treatment of type 2 diabetes mellitus and obesity, but also in cardiovascular disease and for promoting healthy aging [13,19,20]. Recent investigations have shown that exercise enhances the biogenesis of exosomes [21], which carry exerkines to mediate intercellular communication [22] as well as interorgan crosstalk related to exercise [19,20,23]. The exosome release in response to exercise results in a wide range of health benefits including those in type 2 diabetes [24,25], cardiovascular diseases [26,27,28], aging [29] and sarcopenia [30] or immunity [31,32]. We previously demonstrated that 12-weeks of concurrent training (CT) intervention is an effective strategy to induce weight and fat loss with simultaneous reductions of blood pressure and cardiovascular risk (score) and to improve cardiac function in obese men. However, whether this kind of exercise intervention was effective on exosome response was not described in obese populations.

To the best of our knowledge, there is a lack of studies in obese persons addressing the effects of CT intervention (chronic exercise) not only on cardiometabolic health parameters (cardiovascular score), but also on exosome changes in obese populations. Therefore, we hypothesized that the well-known beneficial effect of CT in obese (weight and fat losses or reduction of blood pressure and cardiometabolic risk score) [7] persons could be partially explained by exosomes release. Thus, to study this hypothesis, the present work aimed (I) to analyze the influence of a 12-week CT intervention on exosome cargo modifications in men with obesity and (II) to determine whether changes in exosomes after the intervention were related to changes in cardiometabolic health parameters in our cohorts.

## 2. Materials and Methods

### 2.1. Design and Participants

We performed a 12-week exercise intervention with a parallel-group design strictly following the CONSORT (Consolidated Standards of Reporting Trials) guidelines [33]. The present work is considered a brief research report study that has been conducted in agreement with the last revised ethical guidelines of the Declaration of Helsinki (2013) and has been approved by the Ethics Committee on Human Research (University of León). After providing oral and written informed consent, all participants were randomly assigned into a control group (CG—i.e., no exercise intervention) or a CT group (CT—i.e., aerobic plus resistance training), with this allocation blinded to the assessment staff. All of them were instructed not to modify their nutritional and physical activity routines during the project.

Individuals with obesity—aged from 35 to 55 years—with the absence of additional comorbidities were recruited from the province of Cadiz (Spain) via local media and social networks. Inclusion criteria were (I) men having a body mass index (BMI) higher than 30 kg/m^2^, (II) reporting a sedentary status (i.e., <150 min/week of aerobic physical activity at moderate–vigorous intensity during the last 24 weeks), (III) reporting no significant fluctuations of body weight during the last 3 months and (IV) not suffering any pathological condition which may be aggravated by physical exercise. Exclusion criteria were (I) being under pharmacological intervention for weight loss, (II) reporting an active lifestyle (>150 min/week of aerobic physical activity at moderate–vigorous intensity during the last 24 weeks or participating in a supervised exercise program 2 or more days/week), (III) being under a nutritional program or strategy, (IV) participating in another study. The assessments (i.e., baseline and follow-up) were conducted in the Physical Activity and Exercise Physiology Laboratory at the Faculty of Education Sciences (University of Cádiz, Cádiz, Spain).

### 2.2. Concurrent Training Intervention

A 12-week supervised CT intervention following the physical activity recommendations of the World Health Organization [34] was performed by the participants assigned to the experimental group [7]. In brief, the frequency of exercise sessions was 3 sessions/week with their volume fixed at 60 min. While training intensity was fixed at 60% for 6 weeks and later increased up to 70% of the heart rate reserve for the aerobic part (calculated and adapted each 2 weeks), 6 to 7 of their subjective perceived exertion was the selected intensity resistance training intensity. A total of 3 to 4 sets, including 6–8 exercises, were implemented in a circuit training form, with 1 to 2 min of rest between sets. All exercise sessions began with a dynamic, standardized warm-up, including both activation and a mobility task. A cooling-down protocol was also implemented at the end of all exercise sessions based on the stretching methodology. Free-weight exercises and eight bearings were selected to conduct the resistance training part, including different motor patters (i.e., squat, lateral pull down, dead lift, bench press, etc.). All the training exercise sessions were conducted during the afternoon in the sport facilities located at the Faculty of Education Sciences (University of Cádiz, Cádiz, Spain).

### 2.3. Procedures

Baseline and post-test assessments were conducted on two different days (within the same week). A blood extraction in fasting condition and a medical examination were performed on day 1, while blood pressure, body composition, echocardiography, blood samples, energy metabolism and cardiorespiratory fitness were determined on day 2. Moreover, the dietary intake of 3 24-h recalls and physical activity levels using accelerometry were additionally assessed.

### 2.4. Exosome Isolation

Exosome isolation was performed by modifying the previous protocol [35]. Briefly, after thawing, 1.3 mL of plasma was diluted in an equivalent volume of PBS and centrifuged at 2000× *g* (× *g* expresses the relative centrifugal force) for 30 min at room temperature. The obtained supernatant was centrifuge at 10,000× *g* for 45 min at 4 °C (Sorvall RC-5B Superspeed Centrifuge, DuPont Instruments, Wilmington, DE, USA), and the new supernatant was ultra-centrifuged at 100,000× *g* for 120 min at 4 °C (Optima XL-100K Ultracentrifugue, Beckman Coulter, Brea, CA, USA). The obtained precipitate was resuspended in a volume of PBS equivalent to the initial volume of plasma (1.3 mL) and filtered through 0.2 μm filters [Titan3 PES (Polyethersulfone) Syringe Filters, Thermo Scientific, Waltham, MA, USA]. Subsequently, the filtering was subjected to a new ultracentrifugation at 100,000× *g* at 4 °C (Optima XL-100K Ultracentrifugue, Beckman Coulter). The exosome precipitate was resuspended in 50 μL of 5% SDS-PBS with antiproteases (CompleteTM Mini EDTA-free Protease Inhibitor Cocktail, Roche, Basel, Switzerland) and stored at −80 °C for further analysis.

### 2.5. Western Blot Analysis

DCTM protein assay (Bio-Rad, Hercules, CA, USA) was used to measure the protein content of exosome samples in the microplate reader Synergy HT (BioTek, Winooski, Vermont, USA). Samples containing 40 μg of protein were fractionated by SDS-PAGE on 12% polyacrylamide gels. Then, separated proteins were transferred to PVDF membranes and pre-incubated in 5% non-fat milk for 30 min at 37 °C to block non-specific binding. After that, membranes were incubated overnight at 4 °C with specific primary antibodies. Antibodies against Flotillin-1 (Ref. sc-20066), CD63 (Ref. sc-5275), CD81 (Ref. sc-166029), CD9 (Ref. sc-13118), CD14 (Ref. sc-9150), VDAC1 (Ref. sc-8828) and LAMP-1 (Ref. sc-5570) were purchased from Santa Cruz Biotechnology, Dallas, TX, USA; LAMP2A (Ref. ab18528), HSP70 (Ref. ab2787) and HSP60 (Ref. ab1428) were purchased from Abcam^®^, Cambridge, UK. The bound primary antibody was detected using a horseradish peroxidase (HRP)-conjugated secondary antibody (Dako, Glostrup, Denmark) along with an ECL-HRP kit (Luminol Reagent, Santa Cruz Biotechnology), and blots were exposed to autoradiography films and developed. Finally, the density of the specific bands was quantified with an imaging densitometer (Image J, Bethesda, MD, USA). In the analysis of Western blot bands, the values obtained are usually expressed in optical density (OD) units. However, since each was normalized by CD9, the unit of measurement was cancelled, and the result was expressed as a dimensionless ratio. Therefore, the values would be expressed in “relative units” and not with a specific unit. The presence of the chosen proteins in human exosomes was confirmed using the ExoCarta database [36].

### 2.6. Blood Samples for Determining Cardiometabolic Risk Biomarkers

Blood samples collected from the antecubital vein were taken in a fasted state. All of them were centrifuged (i.e., 4000 rpm during 10 min at 4 °C) and frozen (i.e., at −80 °C). Cardiometabolic health parameters were assessed using routine methods (i.e., spectrophotometry, chemiluminescence assay and enzyme-linked immunosorbent assay) including glycemic profile (i.e., glucose and insulin), lipid profile (i.e., total cholesterol, high-density lipoprotein cholesterol (HDL-C), triglycerides (TGs)), liver profile (i.e., glutamic oxaloacetic transaminase (GOT), glutamic-pyruvic transaminase (GPT), γ-glutamyl transferase (γ-GT)) and inflammatory profile (i.e., C reactive protein (CRP) and leptin). We subsequently calculated HOMA-IR (the homeostatic model assessment of insulin resistance index) [as insulin × glucose/22.5] [37], low-density lipoprotein cholesterol (LDL-C) [as total cholesterol–HDL-C–0.45 × triglycerides], the fatty liver index [as e^(0.953 × loge(TGs) + 0.139 × BMI + 0.718 × loge(γ-GT) + 0.053 × WC − 15.745) × 100] [38] and a cardiometabolic risk Z-score according to the clinical parameters proposed by the IDF (International Diabetes Federation) to diagnose metabolic syndromes including WC, BP, plasma glucose, HDL-C and TGs [39]; for that calculation, the previously-mentioned parameters were standardized (i.e., (value—mean)/standard deviation), multiplying HDL-C value × (−1) to consider a high cardiometabolic risk with greater values. The mean of these 5 standardized values was understood as the cardiometabolic risk Z-score (standard deviation = 1 and mean = 0). 

### 2.7. Blood Pressure

Blood pressure was measured with participants relaxed and sitting in a chair after resting for 5 min. A previously validated Omron M3 device (HEM-7051-E, Kyoto, Japan) was used to assess systolic and diastolic blood pressure, strictly following the European Heart Society’s recommendations [40]. A total of three trials were performed considering the mean value for further analyses: mean blood pressure = systolic blood pressure + (2 × diastolic blood pressure)/3.

### 2.8. Body Composition

A validated stadiometer and scale (SECA 225, Hamburg, Germany) were used to assess height and weight, respectively, with participants barefoot and in light clothing. BMI was subsequently calculated as weight/height^2^. The mid-point between the iliac crest and the rib cage’s bottom was selected to determine waist circumference. Body composition (i.e., fat mass, lean mass and bone mineral content) was determined by electrical bio-impedance (TANITA-MC780MA, Barcelona, Spain) in accordance with the manufacturer’s recommendations.

### 2.9. Echocardiography

All echocardiographic tests were conducted by the same experienced cardiologist—who was blinded to the participants’ study arm—employing an ultrasound system (Sonosite-Edge, Amsterdam, The Netherlands) equipped with a specific transducer. Following the most updated recommendations, cardiac dimensions, volumes and mass were assessed. A pulsed-wave Doppler was used to determine mitral inflow velocities at the end-expiration. Subsequently, left ventricle diastolic function was planned according to the most recent American Society of Ecocardiography consensus guidelines [41]; A wave, E wave, E wave deceleration time and E/A ratio were calculated.

### 2.10. Energy Metabolism

We measured the resting metabolic rate (RMR) in the morning and in a fasted state (10–12 h). The participants were asked to refrain from any physical effort before the test, to avoid moderate/vigorous physical activity (i.e., 24/48 h, respectively), to sleep as usual, to keep their usual nutritional habits and to refrain from alcohol and stimulant intake the day before. Temperature and humidity were strictly controlled (i.e., 20 to 22 °C and 60 to 65%, respectively). RMR was measured with participants in a supine position after resting for 5 min on a bed [42]. A Jaeger MasterScreen CPX^®^ metabolic cart (CareFusion, San Diego, CA, USA) was used to obtain oxygen consumption and carbon dioxide production. A previous gas and volume calibration was systematically conducted before each test in accordance with the manufacturer’ guidelines. All ventilatory data were averaged each 20 s for RMR calculations, discarding the first 10 min. Coefficients of variation were determined for oxygen consumption, carbon dioxide production, respiratory exchange rate and ventilation for each 5 min period, considering those which met the steady state criteria [43]. Previous and well-known equations [44,45] were used to estimate both RMR and substrates oxidation, respectively.

Additionally, we determined the maximal fat oxidation (MFO) during exercise and the intensity that elicits MFO (also known as Fatmax) through a submaximal graded exercise test on a cycloergometer (Lode Excalibur, Groningen, The Netherlands) following standard protocols previously performed and developed [46,47,48].

### 2.11. Cardiorespiratory Fitness

After completing the MFO test and a short break of 3 min, we performed a maximal graded exercise test to determine the maximum oxygen uptake (VO_2max_). The participants started the protocol at the same intensity that they finished the MFO test, increasing 15 W each minute until volitional extenuation, maintaining a cadence of 60–80 rpm. Reaching a respiratory exchange ratio greater than 1.1, attaining an oxygen consumption plateau (i.e., <100 mL/min in the last stages) and reaching a heart rate ±10 beats/min of the theoretical maximal heart rate [49] were considered criteria for achieving VO_2max_.

### 2.12. Physical Activity Levels

Physical activity levels were assessed with accelerometry (ActiGraph GT3X+, ActiGraph, Pensacola, FL, US) during 7 consecutive days (i.e., 24 h). Data processing was conducted with the ActiLife v.6.2.2 software (ActiGraph), excluding registers with <16 h/day and with less than 4 days (1 weekend day included).

### 2.13. Dietary Intake

A certified, trained and qualified dietitian was in charge of the dietary intake assessment through 3 24-h recalls (one weekend day included), all of which were processed by the DIAL^®^ software for Windows (version 3.7.1.0.) energy and macronutrient intakes.

### 2.14. Statistical Analysis

Descriptive data were expressed as mean ± standard deviation. Normality was confirmed using a Shapiro–Wilk test and a visual check of histograms. Unpaired t-tests were implemented (i) to identify potential differences between groups at the baseline and (ii) to determine changes in exosomes between CT vs. CG. Additionally, multilevel mixed analysis was used to analyze the differences in exosome cargo between CT and CG. Net effect (mean difference and standardized mean difference; SMD) was calculated to estimate the clinical relevance of intervention considering the small sample size included in the current study. To examine the relationship between potential changes in exosomes after the intervention and changes in cardiometabolic health parameters (cardiorespiratory fitness, fat mas, blood pressure, Protein C reactive, echocardiography…) in our cohorts, we performed single linear regression analyses, with *p* values equal to or less than 0.05 indicating statistical significance. The regression analyses were applied on calculated variables of the delta values (Post-Pre values; mean differences) of these cardiometabolic health parameters. We used the Statistical Package for Social Sciences (SPSS, v. 22.0, IBM SPSS Statistics, IBM Corporation) for statistical analysis and the GraphPad Prism 5 (GraphPad Software, San Diego, CA, USA) for plot presentations.

## 3. Results

A total of 12 sedentary men with obesity (aged 35–50 years old) were recruited to participate in the present study, attending to >85% of the exercise sessions from baseline to post-test assessment. No significant differences were noted in any outcome at the baseline (Table 1; *p* ≥ 0.1).

Similarly, no significant differences were found in the exosomal levels of β-actin, CD9, CD14, CD63, CD81, Flot-1, GAPDH, HSP60, HSP70, LAMP-1, LAMP-2A and VDAC1 and between CT and CG at both baseline and after the training period (all *p* ≥ 0.14; Table 2).

However, the results from the standardized mean difference (SMD) in Table 2 show a medium to large effect size (from 0.5 to 1.39) of exercise intervention in most of the exosomal changes. 

**Figure 1:**
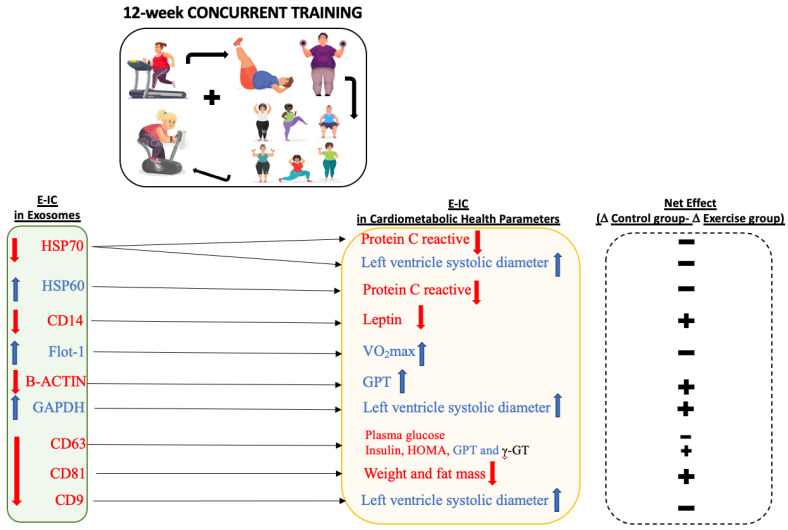
Direction of the exercise-induced changes in exosomes and cardiometabolic health parameters for obese participants in the concurrent training group (*n* = 5). The red and blue colors indicate decreased and increased values after 12 weeks, respectively. Net effect showed the positive or negative sign of differences between changes in the control group minus changes in the exercise group. Negative implies a higher absolute change in the exercise versus control group, while positive means a smaller change in the exercise versus control group. Arrows indicate the relationships found between changes in exosomes and changes in cardiometabolic parameters after 12 weeks in the total sample (*n* = 9).

However, associations between 12 week-induced changes (12W-IC) in exosomes and 12W-IC in cardiometabolic health parameters after the intervention were observed (Table 3). Thus, 12W-IC in HSP70 was positively related to 12W-IC in protein C reactive, while 12W-IC in HSP70 was negatively associated with 12W-IC in left ventricle diastolic diameter (all *p* < 0.031). Similarly, a negative association was found between 12W-IC in HSP60 and 12W-IC in protein C reactive, while a positive relationship was found between 12W-IC in CD14 and 12W-IC in leptin (all *p* < 0.021). Moreover, we obtained a positive relationship of 12W-IC in Flot-1 with 12W-IC in VO_2max_—both in absolute and relative terms (all *p* < 0.040). Interestingly, there was a negative association of 12W-IC in β-actin with 12W-IC in GPT, whereas 12W-IC in GAPDH was positively associated with 12W-IC in left ventricle systolic diameter (all *p* < 0.018). 12W-IC in CD63 was negatively related to 12W-IC in plasma glucose, plasma insulin, GPT and γ-GT (all *p* < 0.05). Furthermore, we also found that 12W-IC in CD81 was positively related to 12W-IC in weight and fat mass, while 12W-IC in CD9 was negatively associated with 12W-IC left ventricle diastolic diameter (all *p* < 0.044). No further associations were noted between 12W-IC in exosome-cargo and 12W-IC in other cardiometabolic health parameters. Moreover, the specific exercise-induced changes in exosomes and cardiometabolic health parameters for obese participants in the concurrent training group (*n* = 5) are shown in Figure 1.

## 4. Discussion

The current study sought to decipher whether a 12-week CT intervention influences exosome cargo in men with obesity and to determine whether changes in exosomes after intervention were related to changes in cardiometabolic health parameters. Although no significant differences between groups were found in the levels of exosomal proteins after the 12-week CT, a medium to large effect size of exercise on most of the exosomal proteins was found. Moreover, associations of changes in exosomes with changes in cardiometabolic health parameters after 12 weeks were revealed.

In general, scientific literature has revealed that exercise promotes the levels of exosome markers CD63, CD81 and Flot-1 [13]. However, to date, there are few studies evaluating the changes in the protein content from exercise-mediated exosomes in humans, and these studies, conducted under acute protocols evaluating the exosome cargo at different sample times, present several inconsistences [50,51]. The study of Brahmer et al. [52], which tried to elucidate the influence of the exosome isolation and phenotyping methods in the determination of the protein variations with exercise, revealed that although most of the proteins evaluated showed the same response, there were different outcomes for some of them depending on the isolation or the phenotyping methods. Thus, the non-significant changes reported here in these exosome protein markers could be influenced by the exosome characterization methodology or sample time. It has also been considered that the duration of the exercise protocol might be a reasonable cause for this lack of variations in the exosome cargo; however, a middle-term resistance exercise that elderly men and women underwent also showed no significant modifications in plasmatic exosome markers in the trained group, although there was a group-by-time interaction effect on the CD63 inflammatory molecule, placing the resistance training as a potential modulator of the inflammageing [53]. Nevertheless, although age might be a key variable whose influence on the results cannot be underestimated, there is a lack of research evaluating the role of exercise-promoted exosome release and cargo modifications in child and elderly populations. It is no less important to mention that much remains to be clarified about the uptake process of the exercise-released exosomes by the rest of the body’s tissues, thus leaving the question as to whether there is no variation in the markers or reuptake in the released exosomes, hindering its detection. In addition, given that medium- and long-term training can generate physiological adaptations, there may be variations in protein load even if there is not a greater number of circulating exosomes. In our case, we did not find changes in the evaluated proteins related to autophagy and mitophagy LAMP-1, LAMP-2A and VDAC1. However, as no previous study has assessed its expression in exosomes in response to exercise, the relevance to describe the lack of changes is highlighted. In addition, multiple cell sources of exercise-promoted exosomes have been identified, so it is still essential to delve into both to determine which tissues are the ones that contribute the most to the pool of circulating exosomes after exercise and which tissues are the ones that exosomes release by exercise and have higher tropism. In addition, it is important to highlight that the effect size findings reveal a medium to large effect of the intervention on those exosomes, although caution in their interpretation needs be reminded.

Interestingly, our outcomes reveal several associations between 12W-IC and exosome-carried proteins that could highlight the physiological or clinical implication of exercise-released exosomes on cardiometabolic health parameters of obese participants. Maximal oxygen consumption (VO_2max_) is a clinical predictor for cardiovascular diseases and all-cause mortality [54] and is related to BMI [55]. Endurance training has proven to increase the total mitochondrial volume, along with VO_2max_ [56], and improve BMI [57]. Here, we present, for the first time, a positive relationship between 12W-IC in Flot-1 and CD8, with changes in VO_2max_ and weight and fat mass, respectively. Although there is no previous published evidence of such relationships between these variables and exosomal protein content, recent studies have shown associations between some exosomal miRNAs and VO_2max_ as indicators of physiological adaptations to physical activity [58]. This could support the association between changes in VO_2max_ and changes in exosomes found in this study. In fact, it would be plausible that fitness level and other individual physical characteristics, such as sex or BMI, could affect the exosomal release and cargo modifications in response to exercise [59]. Of relevance, our previous work [7] on these participants reported effectiveness on the training program for losing weight and fat mass.

On the other hand, elevated levels of extracellular HSP70 have been described in obese persons; however, the behavior of this protein in response to exercise is not clear since both a decreased [60] and increased [61] release have been observed after exercise, which might depend on the exercise intensity [61], among other factors. In our data, despite no statistically significant difference between control and exercise groups, the mean value of HSP70 increased in control groups and decreased in exercise groups after 12 weeks (Figure 1), with a large effect size supporting this finding. Moreover, changes in exosomal HSP70 were positively associated with protein C reactive and negatively associated with changes in left ventricular diastolic diameter, which is a risk indicator in coronary artery disease [62]. In fact, our previous work demonstrated an improvement in the left ventricular end-diastolic diameter after 12 weeks of CT [7]. Thus, all this together suggests that CT exercise could decrease levels of extracellular HSP70 in parallel to a reduction in protein C reactive and improve cardiac structure (left ventricular). Interestingly, it is aligned with evidence that reported a close relationship between left ventricular dysfunction and metabolic abnormalities [63]. Extracellular HSP70 has been proven to play roles in cardiovascular (patho) physiology. Thus, circulating HSP72 has been found to correlate significantly with left ventricular end-diastolic dimension values in patients with idiopathic left ventricular dysfunction [64]. Moreover, HSP70 plasma levels were enhanced in hypertension during pregnancy [65] and were directly associated with the prognosis of heart failure [66] as well as with increased risk [67] and severity of acute coronary syndromes [68].

Human monocyte differentiation antigen CD14 is a pattern recognition receptor that acts as a co-receptor of TLR4, increasing its activity. However, apart from its proinflammatory canonical role, multiple functions in pathogenesis have arisen, which could be due to its wide range of ligands such as HSP70 or leptin [69]. CD14 has been observed to be upregulated in monocytes, adipocytes and whole adipose tissue [70] and has been presented as a predictor of subclinical inflammatory obesity [15], but little is known about the role of soluble CD14, especially the exosomal form. Here, we present a non-statistically significant reduction (but clinically relevant; medium effect size) in exosomal CD14, a positive relationship between 12W-IC and CD14 and a decrease in leptin levels (Figure 1). These findings are in line with the actual scientific consensus that exercise decreases leptin levels [71,72] in obese individuals [73,74], suggesting that it could be mediated, at least in part by a decrease in exosomal CD14. The link between leptin and CD14 has also been supported by others [75] that found leptin induces CD14/TLR4 activation by the JAK2-STAT3 signaling pathway to promote obesity-related osteoarthritis. 

Another set of relevant findings from our study showed negative relationships of 12W-IC in CD63 with 12W-IC in plasma glucose, plasma insulin, GPT and γ-GT, along with a positive association of 12W-IC in CD81 with 12W-IC in weight and fat mass. Although there is a lack of knowledge about the specific role of exosomal tetraspanins in cardiovascular injuries, vascular smooth muscle cell calcification has been proven to be mediated by regulated exosome secretion enriched with the tetraspanins CD9, CD63 and CD81 [76]. However, more research is needed to confirm our results and to clarify the specific role of 12W-IC in CD63 and CD81 in relation to cardiometabolic risk factors such as glucose, insulin or fat mass levels.

In addition, the present study presented several limitations. Since the obesity pathological state may influence the changes of the exosome cargo in response to exercise, upcoming investigations should include a normal-weight control group. On the other hand, only exosomal protein levels were evaluated, so future research performing particle counting in addition to using different isolation and phenotyping methodologies is needed in order to broaden knowledge about exosomal modifications in response to different concurrent exercise protocols in obesity. Moreover, a different approach focused on exerkines released from adipose tissue could have provided additional information on the beneficial effect of exercise and the consequent release of exosome in obese people. Finally, the small sample size of participants with valid data for all the selected variables constitute a statistical limitation in terms of power; however, as revealed by SMD calculated in Table 2 for estimating the clinical relevance of intervention, the small sample size of the current study provides changes that are biologically/clinically relevant. Additionally, some strengths merit the need to be mentioned. This is one of the first studies in obese persons addressing the chronic effects of CT intervention not only on cardiometabolic health parameters, but also on exosome changes in this population. Moreover, the assessment of such a large number of variables and dimensions in relation with exosomes within the context of CT intervention provides extra added value.

Taking all these together, these findings provide a clinical perspective from which implementing exercise programs of CT in obese populations is not only effective and useful in terms of classical cardiometabolic health parameters, but also in the activation of exosomal release that could explain these health benefits and even be mediating others still unknown. Therefore, this study provides an added value to the current scientific evidence by means of showing that supervised CT programs have powerful health effects in obese populations and, of particular interest, that these benefits are also at an exosomal level. Nevertheless, more research is needed to better know to which extent this exosomal response can mediate health benefits and to corroborate the results in a larger sample size.

In conclusion, the current investigation revealed associations between exercise-driven variations in some exosomal proteins (CD14, CD63, CD81, Flot-1, GAPDH, HSP60, HSP70) with modifications in body composition (weight and fat mass), markers of inflammation (CRP), as well as cardiorespiratory (VO_2max_) and cardiometabolic (left ventricle diastolic and systolic diameters, leptin, plasma glucose, insulin, GPT and γ-GT) health parameters; thus, exercise-promoted exosomes in obese populations could provide a potential therapeutic application via the role of intercellular communication. Nevertheless, further studies are needed to establish a cause–effect link between these associations in response to training.

## Figures and Tables

**Table 1:** Baseline descriptive characteristics of the study subjects.

	Control Group (*n* = 4)	Concurrent Training Group (*n* = 5)
Age (years)	43.7 (6.1)	41.3 (4.4)
Anthropometry and body composition		
Weight (kg)	101.4 (12.9)	97.5 (15.4)
Height (cm)	176.4 (5.2)	173.9 (8.7)
Body mass index (kg/m^2^)	32.5 (3.0)	32.1 (3.6)
Waist circumference (cm)	108.0 (6.1)	105.4 (9.4)
Fat mass (kg)	28.5 (7.3)	27.8 (6.6)
Lean mass (kg)	69.3 (6.9)	66.3 (9.3)
Blood pressure		
Systolic blood pressure (mm Hg)	131.5 (16.9)	131.4 (22.0)
Diastolic blood pressure (mm Hg)	83.2 (6.3)	86.6 (13.9)
Mean blood pressure (mm Hg)	99.3 (9.2)	101.5 (16.5)
Glycaemic profile		
Plasma glucose (mg/dL)	101.5 (3.9)	94.8 (9.1)
Plasma insulin (UI/mL)	13.9 (4.2)	8.6 (2.0)
HOMA-IR	3.47 (1.02)	2.00 (0.52)
Lipid profile		
Total cholesterol (mg/dL)	216.0 (59.8)	198.0 (22.6)
HDL-C (mg/dL)	48.8 (9.6)	40.8 (5.0)
LDL-C (mg/dL)	145.0 (50.3)	130.8 (22.0)
Triglycerides (mg/dL)	112.0 (53.9)	131.4 (70.5)
Liver function		
GOT (IU/L)	29.3 (8.5)	21.6 (8.1)
GPT (IU/L)	44.0 (27.5)	22.0 (7.2)
γ-GT (IU/L)	68.5 (75.2)	73.3 (62.7)
Other biochemical parameters		
Protein C reactive (mg/dL)	11.7 (18.1)	8.2 (9.4)
Leptin (ng/mL)	19.8 (5.1)	21.0 (6.4)
Cardiorespiratory fitness		
VO_2max_ (ml/min)	2596.3 (472.2)	2620.3 (573.3)
VO_2max_ (ml/kg/min)	25.6 (2.3)	27.4 (7.4)
Echocardiography		
Cardiac mass (g)	207.4 (32.0)	192.6 (27.8)
Ejection fraction (%)	65.8 (6.2)	66.2 (5.7)
LV end diastolic diameter (mm)	52.5 (5.2)	52.6 (3.2)
LV end systolic diameter (mm)	26.3 (4.0)	33.4 (3.6)
LV end systolic volume (ml)	21.5 (10.3)	22.4 (2.4)
E wave (cm/s)	87.3 (12.5)	70.2 (20.8)
A wave (cm/s)	55.8 (3.7)	65.8 (17.4)
E/A	1.57 (0.26)	1.07 (0.13)
E wave deceleration time (ms)	205.0 (20.4)	261.0 (43.8)

Abbreviations: HOMA, homeostasis model assessment; HDL-C, high-density lipoprotein cholesterol; LDL-C, low-density lipoprotein cholesterol; GOT, glutamic oxaloacetic transaminase; GPT, glutamic-pyruvic transaminase, γ-GT, γ-glutamyl transferase; VO_2max_, maximal oxygen uptake; LV, left ventricle.

**Table 2:** Changes in exosome release after a 12-week intervention among the control and concurrent training groups.

	Control Group (*n* = 4)			Concurrent Training Group (*n* = 5)			Net Effect	
	Baseline Mean (SD)	After 12 Weeks Mean (SD)	Δ (SE)	Baseline Mean (SD)	After 12 Weeks Mean (SD)	Δ (SE)	Mean Difference (95% CI)	Standardized Mean Difference (95% CI)
LAMP-1 (RU)	3,538,140.1 (138,437.8)	4,662,487.6 (3,259,796.9)	1,124,347.5 (1,678,798.8)	6,376,794.4 (2,524,368)	4,409,439.5 (3,869,631)	−1,967,354.9 (1,176,955.3)	3,091,702.4 (−2,001,411.9, 8,184,816.7)	1.04 (−0.36, 2.44)
LAMP-2A (RU)	2,888,610.7 (1,463,067.7)	2,198,326.8 (1,213,501.9)	−690,283.9 (1,130,472.5)	2,459,521.9 (1,726,292.9)	3,763,442.9 (3,202,342.4)	1,303,921 (1,235,228.1)	−1,994,204.9 (−5,956,063.6, 1,967,653.8)	−0.78 (−2.14, 0.58)
HSP70 (RU)	3,129,771.2 (611,074.9)	4,309,378.3 (2,413,580.4)	1,179,607.1 (1,004,876.6)	4,565,930.3 (1,454,915.8)	3,382,425.5 (869,688.3)	−1,183,504.7 (479,725.9)	2,363,111.8 (−631,925.8, 5,358,149.4)	1.53 (−0.04, 3.02)
HSP60 (RU)	3,348,621 (2,127,044.5)	3,565,455.3 (679642.8)	216834.3 (1,333,310)	2,893,043.2 (2,090,405.6)	4,219,214.8 (2,215,688.5)	1,326,171.7 (1,070,853.8)	−1,109,337.4 (−5,263,234.9, 3,044,560.2)	−0.44 (−1.77, 0.89)
CD14 (RU)	2,976,028.6 (1,155,800.9)	3,357,159.3 (1704612)	381130.7 (485,199.3)	3,905,096.1 (1,768,269.5)	3,623,449.7 (1,714,922.5)	−281,646.4 (611,570.1)	662,777.1 (−1,185,935.5, 2,511,489.6)	0.55 (−0.79, 1.89)
Flot-1 (RU)	3,339,068.3 (1,856,409.4)	5,912,644.2 (1053525)	2,573,575.9 (619,043)	4,667,901.4 (3,443,030.5)	5,923,898.6 (2,682,591.2)	1,255,997.2 (694,489.7)	1,317,578.7 (−882,689, 3,517,846.5)	0.92 (−0.46, 2.31)
B-ACTIN (RU)	2,343,284.8 (1,263,330.2)	3,141,978 (1,417,101.6)	798,693.2 (435,468)	2,935,534.3 (1,949,914.3)	2,039,544.3 (875,454.4)	−895,990 (737,123.1)	1,694,683.2 (−379,143.8, 3,768,510.2)	1.24 (−0.20, 2.67)
GAPDH (RU)	2,215,793.1 (1,214,415.7)	4,036,692.8 (995245.3)	1,820,899.7 (676,474)	2,022,621.8 (1,222,361.4)	2,805,584.7 (1,311,539.3)	782,963 (536,431)	1,037,936.7 (−1,063,093.6, 3,138,967)	0.82 (−0.55, 2.19)
VDAC1 (RU)	2,360,209.4 (592,894.3)	4,284,494.8 (1,180,678.8)	1,924,285.4 (500,062.3)	4,253,649.8 (3,249,022.7)	3,341,431.8 (1,180,400.6)	−912,218 (1,146,500.7)	2,836,503.4 (−307,544.7, 5,980,551.4)	1.39 (−0.08, 2.85)
CD63 (RU)	5,811,173.1 (2,704,553.8)	4,030,302.7 (2,108,586.8)	−1,780,870.4 (1,096,623.9)	5,547,351 (2,466,183.1)	3,235,053.4 (2,490,041)	−2,312,297.6 (716,172.1)	531,427.2 (−2,766,527.5, 3,829,381.9)	0.28 (−1.04, 1.60)
CD81 (RU)	3,779,431.7 (1,366,587.2)	6,389,106.8 (1,339,581.9)	2,609,675.1 (323,006.5)	5,669,999.2 (3,019,804.5)	5,199,976 (2,259,769.5)	−470,023.2 (520,670.7)	3,079,698.3 (1,603,159.5, 4,556,237.1)	1.19 (−0.53, 2.85)
CD9 (RU)	2,372,863.1 (336,531.5)	3,638,204.5 (1,370,077.8)	1,265,341.5 (815,938.4)	3,892,886.6 (159,3471)	2,455,290.1 (1,781,346.2)	−1,437,596.5 (1,282,990.8)	2,702,938 (−952,067, 6,357,943)	1.12 (−0.30, 2.53)

Analyses were performed using multilevel mixed analysis, including the group as a fixed variable (control group vs. exercise group), adjusted for the baseline values of the specific outcome assessed. RU, relative units.

**Table 3:** Association between changes in exosomes and changes in cardiometabolic health parameters after the intervention in men with obesity.

	LAMP-1 (RU)	LAMP-2A (RU)	HSP70 (RU)	HSP60 (RU)	CD14 (RU)	Flot-1 (RU)	Β-ACTIN (RU)	GAPDH (RU)	VDAC1 (RU)	CD63 (RU)	CD81 (RU)	CD9 (RU)
**Anthropometry and body composition**												
Weight (kg)	0.114	−0.261	0.509	0.127	−0.005	0.497	0.436	0.318	0.647	−0.298	**0.696**	0.448
Waist circumference (cm)	−0.041	−0.321	0.427	−0.014	0.102	0.124	0.33	−0.036	0.535	0.183	0.518	0.449
Fat mass (kg)	0.212	−0.313	0.568	0.083	0.042	0.425	0.439	0.418	0.661	−0.337	**0.741**	0.497
Lean mass (kg)	−0.099	−0.123	0.323	0.204	−0.074	0.582	0.395	0.069	0.543	−0.145	0.528	0.298
**Blood pressure**												
Systolic blood pressure (mm Hg)	0.503	0.098	0.403	−0.104	0.351	0.222	0.171	0.134	0.227	−0.345	0.573	0.133
Diastolic blood pressure (mm Hg)	0.490	−0.076	0.482	−0.059	0.331	0.297	0.309	0.296	0.38	−0.341	0.677	0.284
Mean blood pressure (mm Hg)	0.497	−0.023	0.46	−0.073	0.339	0.275	0.268	0.247	0.334	−0.344	0.649	0.239
**Glycaemic profile**												
Plasma glucose (mg/dL)	0.324	0.224	0.379	−0.187	0.04	−0.078	−0.218	−0.037	0.133	−0.537	0.423	0.031
Plasma insulin (UI/mL)	0.322	0.244	0.003	−0.001	−0.182	−0.016	−0.388	0.326	−0.25	**−0.760**	−0.024	−0.259
HOMA-IR	0.304	0.223	0.019	0.015	−0.182	−0.047	−0.38	0.359	−0.216	**−0.765**	−0.006	−0.226
**Lipid profile**												
Total cholesterol (mg/dL)	0.105	0.569	0.062	−0.067	−0.247	−0.006	−0.602	−0.425	−0.247	−0.691	−0.013	−0.395
HDL-C (mg/dL)	0.122	−0.033	0.504	−0.387	−0.204	−0.439	−0.384	−0.459	0.217	−0.456	0.155	0.193
LDL-C (mg/dL)	−0.16	0.696	−0.119	0.227	−0.14	0.03	−0.492	−0.361	−0.217	−0.48	0.006	−0.42
Triglycerides (mg/dL)	0.487	0.228	0.184	−0.415	−0.309	0.077	−0.522	−0.264	−0.311	−0.746	−0.097	−0.323
**Liver function**												
GOT (IU/L)	0.125	−0.186	0.443	−0.142	−0.322	−0.282	−0.238	0.308	0.337	−0.666	0.247	0.295
GPT (IU/L)	0.223	0.554	−0.063	0.014	0.026	−0.623	**−0.759**	−0.239	−0.498	**−0.803**	−0.212	−0.426
γ-GT (IU/L)	−0.143	0.548	−0.102	0.279	−0.208	−0.445	−0.684	−0.074	−0.256	**−0.778**	−0.147	−0.328
**Other biochemical parameters**												
Protein C reactive (mg/dL)	0.693	−0.715	**0.800**	**−0.917**	0.362	0.128	0.588	−0.251	0.541	0.469	0.616	0.650
Leptin (ng/mL)	0.662	0.050	0.264	−0.191	**0.760**	−0.512	0.082	0.279	−0.059	−0.160	0.448	0.129
**Cardiorespiratory fitness**												
VO_2_max (ml/min)	−0.480	−0.392	0.227	0.257	−0.511	**0.740**	0.435	0.204	0.645	0.083	0.350	0.410
VO_2_max (ml/kg/min)	−0.555	−0.369	0.170	0.283	−0.491	**0.730**	0.444	0.165	0.623	0.197	0.310	0.392
**Echocardiography**												
Cardiac mass (g)	−0.128	0.719	−0.296	0.000	0.097	−0.596	−0.633	−0.574	−0.537	−0.173	−0.373	−0.527
Ejection fraction (%)	0.082	−0.286	0.359	−0.171	0.121	0.216	0.377	0.275	0.398	0.353	0.393	0.411
LV end diastolic diameter (mm)	−0.673	0.635	**−0.804**	0.324	−0.438	0.204	−0.458	−0.562	−0.634	0.160	−0.728	**−0.784**
LV end systolic diameter (mm)	0.236	−0.447	0.027	0.180	0.210	0.427	0.570	**0.812**	0.267	0.200	0.293	0.307
LV end systolic volume (ml)	−0.267	0.017	0.085	−0.390	−0.674	−0.241	−0.524	−0.666	−0.146	−0.233	−0.425	−0.143
E wave (cm/s)	0.126	−0.204	0.525	−0.660	−0.173	−0.313	−0.178	−0.624	0.174	0.055	0.044	0.255
A wave (cm/s)	−0.037	−0.005	0.314	0.388	0.410	0.295	0.471	0.239	0.563	−0.039	0.717	0.386
E/A	0.035	−0.039	0.039	−0.667	−0.414	−0.389	−0.484	−0.642	−0.346	0.116	−0.533	−0.188
E wave deceleration time (ms)												

Linear regression analysis coefficient (B) is expressed for each association. Bold numbers denote statistically significant association (*p* ≤ 0.05).

## Data Availability

The datasets and material generated and analyzed for this study are available upon request to interested researchers.

## Abbreviations

HOMA, homeostasis model assessment; HDL-C, high-density lipoprotein cholesterol; LDL-C, low-density lipoprotein cholesterol; GOT, glutamic oxaloacetic transaminase; GPT, glutamic-pyruvic transaminase, γ-GT, γ-glutamyl transferase; VO_2max_, maximal oxygen uptake; LV, left ventricle; RU, relative units.
